# Supplementary figures and images for: Plasma Membrane Calcium ATPase Regulates Stoichiometry of CD4+ T-Cell Compartments
Source: Front Immunol. 2021 May 21;12:687242. doi: 10.3389/fimmu.2021.687242 (PMC8175910; doi:10.3389/fimmu.2021.687242)

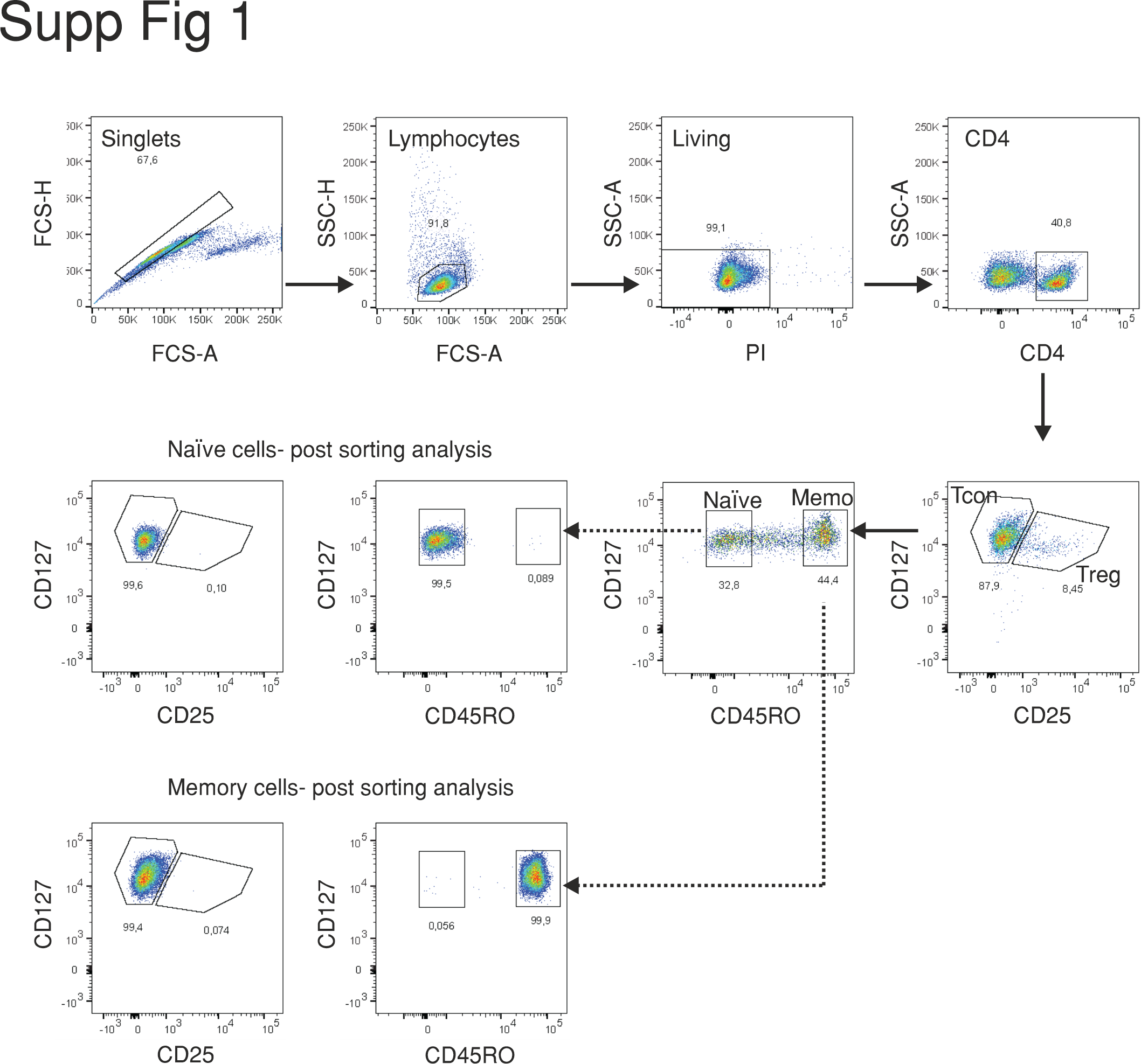

Supplement: Supplementary Figure 1 — Sorting strategy of naïve and memory cells Representative flow cytometry images showing the isolation FACSorting strategy of human naïve (CD4+CD127highCD25-CD45RO-) or memory (CD4+CD127highCD25-CD45RO+) T cells using FACSAria III (BD). [file Image_1.tif]
