# Supplementary material for: Plasma Membrane Calcium ATPase Regulates Stoichiometry of CD4+ T-Cell Compartments
Source: Front Immunol. 2021 May 21;12:687242. doi: 10.3389/fimmu.2021.687242 (PMC8175910; doi:10.3389/fimmu.2021.687242)
Supplement: Supplementary file 5 [file DataSheet_1.pdf]

Supplemental table 1: List of transcription factors identified by in silico analysis

Trancription factor/ Gene ID	TSS 500	Cell types	TSS 1000	Cell types	TSS 5000	Cell types	TSS 10000	Cell types
YY1 [7528]	170	13	257	13	653	13	905	13
JUN [3725]	138	7	182	7	347	7	543	11
SP1 [6667]	101	7	152	7	355	8	496	8
E2F1 [1869]	81	4	110	4	205	4	298	5
FOS [2353]	78	5	144	5	345	5	582	6
ETS1 [2113]	77	8	94	8	213	8	322	8
MAZ [4150]	73	6	111	6	244	6	317	6
TBP [6908]	70	5	102	5	189	5	276	5
IRF1 [3659]	66	3	101	3	191	3	281	4
ATF3 [467]	63	6	93	6	215	6	336	11
GATA1 [2623]	54	3	102	3	214	3	367	4
RXRA [6256]	54	3	78	5	178	5	235	6
GATA2 [2624]	42	4	65	5	149	6	273	9
PAX5 [5079]	38	5	51	6	93	6	138	7
USF2 [7392]	38	5	64	6	206	6	313	6
IRF2 [3660]	34	2	53	2	88	2	112	2
MYB [4602]	33	3	55	3	87	3	106	3
NFIC [4782]	30	2	45	2	84	2	121	2
ELK1 [2002]	29	5	49	5	145	5	226	5
SRF [6722]	29	2	36	2	107	3	147	3
LEF1 [51176]	27	2	41	2	106	2	183	2
CEBPA [1050]	24	4	46	6	79	6	89	7
HNF4A [3172]	24	2	40	3	119	4	136	5
WT1 [7490]	23	1	36	1	76	1	110	1
NFATC1/T2 [4772]	16	2	18	2	34	2	42	2
MEF2A [4205]	13	2	17	2	39	2	59	2
ETS2 [2114]	9	2	10	2	28	2	63	2
AHR [196]	8	2	9	2	15	2	26	3
HNF1A [6927]	8	1	14	1	20	1	37	1
NFKB1 [4790]	2	1	2	1	8	1	34	2
STAT4 [6775]	2	1	2	1	4	1	12	1
VDR [7421]	2	1	12	3	17	4	23	4

List of transcription factors detected in all three algorithms used for prediction of potential transcription factors regulating ATP2B4 in (Fig. 7A), see results and material and methods sections for detailed description of the used algorithms. Scores are calculated based on the probability of binding to a sequence of 500, 1000, 5000 and 10000 nucleotides long from the starting transcription site (TSS) in the *ATP2B4* gene with the corresponding number of cell types in which transcription was tested

Supplemental Table 2: List of antibodies used for flow cytometry

Antigen	Fluorophore	Supplier	Order number
CD4	PerCP-Cy5.5	Biolegend	300530
CD127 (IL-7Ra)	Alexa Fluor® 700	Biolegend	351344
CD25	FITC	Biolegend	302604
CD45RO	PE-Cy7	Biolegend	304230
CD197(CCR7)	Alexa Fluor® 647	Biolegend	353218
CD45RA	PE	Biolegend	304108
CD62L	Pacific Blue	Biolegend	304826
CD69	Pacific Blue	Biolegend	310919
CD154	APC	Biolegend	310809

List of antibodies used for flow cytometry
